# Perturbed autophagy and DNA repair converge to promote neurodegeneration in amyotrophic lateral sclerosis and dementia

**DOI:** 10.1093/brain/awy076

**Published:** 2018-03-23

**Authors:** Callum Walker, Sherif F El-Khamisy

**Affiliations:** 1 Krebs Institute, Department of Molecular biology and biotechnology, University of Sheffield, UK; 2 The Institute of Cancer Research, London, UK; 3 Center for Genomics, Helmy Institute for Medical Sciences, Zewail City of Science and Technology, Giza, Egypt

**Keywords:** R-loops, autophagy, DNA repair, genomic instability, neurodegeneration

## Abstract

Maintaining genomic stability constitutes a major challenge facing cells. DNA breaks can arise from direct oxidative damage to the DNA backbone, the inappropriate activities of endogenous enzymes such as DNA topoisomerases, or due to transcriptionally-derived RNA/DNA hybrids (R-loops). The progressive accumulation of DNA breaks has been linked to several neurological disorders. Recently, however, several independent studies have implicated nuclear and mitochondrial genomic instability, perturbed co-transcriptional processing, and impaired cellular clearance pathways as causal and intertwined mechanisms underpinning neurodegeneration. Here, we discuss this emerging paradigm in the context of amyotrophic lateral sclerosis and frontotemporal dementia, and outline how this knowledge paves the way to novel therapeutic interventions.

## Teaser

Accumulation of DNA breaks has been linked to several neurological disorders. However, only recently have links started to emerge between DNA damage, perturbed co-transcriptional processing, and impaired cellular clearance pathways as causal and intertwined mechanisms underpinning neurodegeneration. Walker and El-Khamisy discuss these new links with a focus on ALS.

## Genomic instability: a neuronal perspective

The ability to maintain genomic integrity represents a major challenge facing cells. A variety of exogenous and endogenous agents can induce DNA damage (Ciccia and Elledge, 2010) with each cell estimated to process ∼10^13^ lesions each day (Lindahl and Barnes, 2000). Many normal cellular processes induce DNA damage. For example, reactive oxygen species, a natural by-product of cellular respiration, oxidize nucleic acids causing DNA damage (Valko *et al.*, 2006). The faulty action of DNA processing enzymes, such as DNA ligases and topoisomerases, induce DNA lesions (El-Khamisy *et al.*, 2005; Ahel *et al.*, 2006). Furthermore, the fundamental cellular process of transcription leads to additional DNA damage due to the formation of DNA/RNA hybrids (R-loops) (Skourti-Stathaki and Proudfoot, 2014). The cell is therefore under constant pressure to maintain genomic stability.

The DNA damage response comprises multiple distinct repair pathways, each taking precedence in response to different forms of damage (Ciccia and Elledge, 2010). More often, damage to the DNA duplex is restricted to one strand of the phosphodiester backbone (single-stranded breaks), but can also occur on both strands (double-stranded breaks, DSBs). Though less common, DSBs are highly mutagenic and cytotoxic (Khanna and Jackson, 2001). To address this threat, cells have evolved a high fidelity DSB repair mechanism, homologous recombination, which utilizes sister chromatids as a template for polymerase rectification (Hartlerode and Scully, 2009). During G2 and S phase, cells can utilize homologous recombination to perform error-free DSB repair. Post-mitotic neurons, however, are intrinsically homologous recombination-deficient (El-Khamisy, 2011; Rulten and Caldecott, 2013), relying on error-prone non-homologous end-joining to repair DSBs.

DNA repair defects often present themselves clinically as neurological disease (Madabhushi, *et al.*, 2014). For example, DSB repair dysfunction leads to the neurodegenerative diseases, ataxia telangiectasia (Savitsky *et al.*, 1995) and ataxia telangiectasia-like disorder (Stewart *et al.*, 1999). Single-stranded break repair defects are also associated with a number of neurological diseases, including cerebellar ataxias (Moreira *et al.*, 2001, 2004; Takashima *et al.*, 2002; Hoch *et al.*, 2017), and has been implicated in Cockayne syndrome (Nance and Berry, 1992) and xeroderma pigmentosum (Kraemer, *et al.*, 1987). More recently, defective DNA repair has been characterized as an important pathological process in motor neuron disease, otherwise known as amyotrophic lateral sclerosis (ALS).

## Amyotrophic lateral sclerosis

ALS is a heterogeneous disease characterized by the loss of motor neurons. While sporadic in ∼90% of patients, 10% are familial and are caused by mutations in myriad of genes. ALS-causing mutations often occur within genes associated with RNA processing or cellular degradation (Hardiman *et al.*, 2017). Interestingly, ALS patients can develop frontotemporal dementia (FTD) in unison and the two diseases appear to co-exist in a single disease spectrum (Couratier *et al.*, 2017). Mutations in the *TARDBP* gene, encoding the RNA processing factor TAR DNA-binding protein-43 (TDP-43), are known to cause ALS and FTD (Kabashi *et al.*, 2008). In addition, mutations in other RNA processing factors, such as *FUS* (encoding fused in sarcoma) (Kwiatkowski *et al.*, 2009; Vance *et al.*, 2009), *SETX* (encoding senataxin) (Chen *et al.*, 2004), and *HNRNPA*/*B* (encoding heterogeneous nuclear ribonucleoprotein A/B, hnRNPAB) (Kim *et al.*, 2013) also cause ALS. Mutations in the *SMN1* gene (encoding the core spliceosome component survival of motor neuron, SMN), lead to a severe form of juvenile motor neuron disease called spinal muscular atrophy (Melki *et al.*, 1990). Thus, motor neuron disease is frequently associated with mutations in genes involved with RNA processing.

As well as RNA processing dysfunction, ALS is strongly associated with defects in autophagy and the ubiquitin proteasome system (UPS) (Majcher *et al.*, 2015; Hardiman *et al.*, 2017), which are the primary degradation pathways available to mammalian cells. Mutations in *VCP* (encoding valosin-containing protein, also known as p97 or Cdc48), cause ALS, FTD and a related syndrome known as inclusion body myopathy with Paget disease of bone and frontotemporal dementia (IBMPFD) (Watts *et al.*, 2004; Johnson *et al.*, 2010). VCP functions as a central component of the UPS (Meyer and Weihl, 2014), and is also implicated in autophagy (Majcher *et al.*, 2015). Alike VCP, mutations in the *SQSTM1* gene (encoding p62, also known as sequestosome-1) cause ALS, FTD and IBMPFD (Fecto *et al.*, 2011; Rea *et al.*, 2014). P62 is important for the process of ubiquitin-mediated autophagy (Pankiv *et al.*, 2007; Katsuragi *et al.*, 2015), and ALS-causing mutations perturb this process (Goode *et al.*, 2016). Mutations in the *UBQLN2* gene (encoding for ubiquillin-2), which acts as a dual regulator of autophagy and the ubiquitin proteasome system (Ceballos-Diaz *et al.*, 2015; Osaka *et al.*, 2016), also cause ALS (Deng *et al.*, 2011). Taken together, these examples demonstrate that genetic perturbations in cellular clearance pathways are also associated with ALS.

A hexanucleotide repeat expansion contained within the first intron of the *C9orf72* gene, was recently discovered to be the most common genetic cause of ALS and FTD (DeJesus-Hernandez *et al.*, 2011; Renton *et al.*, 2011). The C9orf72 protein is now characterized as an autophagy coactivator (Webster *et al.*, 2016; Yang *et al.*, 2016). C9orf72 protein levels are also reduced in patient tissue (DeJesus-Hernandez *et al.*, 2011; Belzil *et al.*, 2013; Waite *et al.*, 2014), suggesting a possible role for haploinsufficiency and defective autophagy in C9orf72-ALS. In addition, RNA molecules transcribed from the C9orf72 expansion form nuclear RNA foci (Zu *et al.*, 2013; Cooper-Knock *et al.*, 2014). Intronic C9orf72 RNAs are also exported into the cytoplasm, and undergo repeat associated non-ATG dependent (RAN)-translation (Ash *et al.*, 2013; Zu *et al.*, 2013), producing five dipeptide repeat proteins. C9orf72 expansions are associated with aberrant nucleocytoplasmic transport (Freibaum *et al.*, 2015; Jovicic *et al.*, 2015; Zhang *et al.*, 2015*a*), dysfunctional RNA processing (Lee *et al.*, 2013; Cooper-Knock *et al.*, 2014; Prudencio *et al.*, 2015; Yin *et al.*, 2017), and impaired cellular clearance mechanisms (Zhang *et al.*, 2014; Gao *et al.*, 2017; Gupta *et al.*, 2017; Ramesh and Pandey, 2017). It is thought that some contribution of these factors conspires to drive neurodegeneration in C9orf72-ALS.

In line with the genetic aetiology of ALS, CNS tissues from around 97% of ALS patients, including both sporadic and familial cases, display the mislocalization and aggregation of TDP-43 (TDP-43 proteinopathy) (Neumann *et al.*, 2006; Ling *et al.*, 2013). Given the role of TDP-43 in nuclear RNA processing pathways (Scotter *et al.*, 2015), TDP-43 mislocalization likely represents a loss of its nuclear function and a defect in RNA processing. Sporadic and familial ALS patient tissues also display a second disease hallmark, the accumulation and aggregation of p62. P62 binds to misfolded protein aggregates, targeting them for degradation by autophagy (Bjørkøy *et al.*, 2005). P62 itself is degraded during autophagy (Ichimura *et al.*, 2008) and the accumulation of p62 in ALS patients likely indicates a defect in this process. Understanding the pathological consequences of these two disease hallmarks will likely unlock the door to novel treatment options for ALS patients.

### ALS-linked RNA processing factors are guardians of the genome

**Figure 1:**
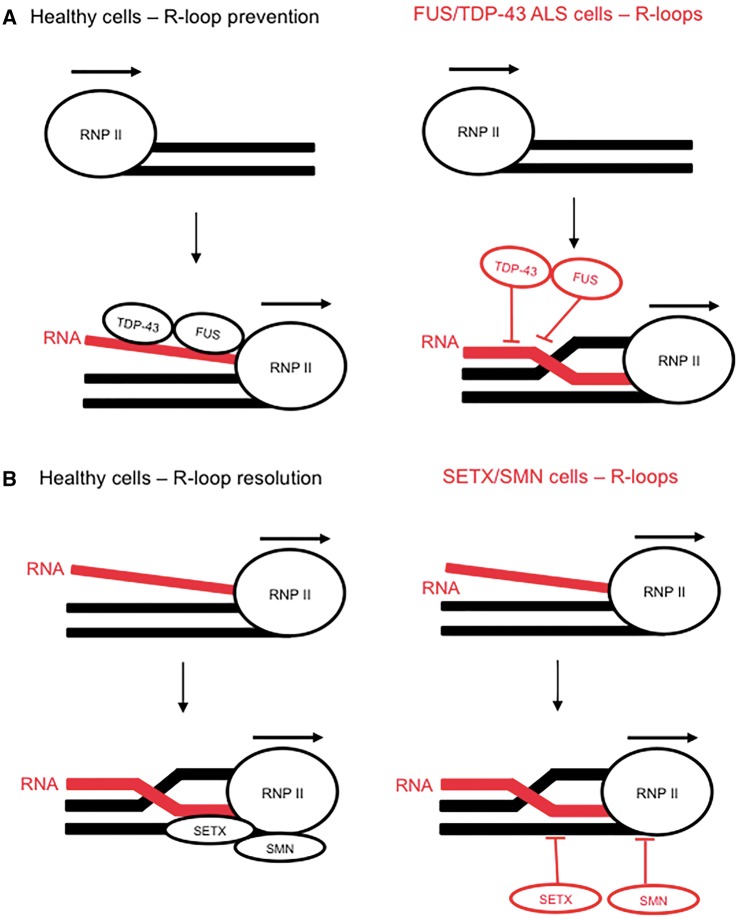
**ALS-linked RNA processing factors are guardians of the genome**. (**A**) Several factors associated with RNA processing, including FUS and TDP-43, are required to maintain R-loop homeostasis. Their depletion leads to R-loop-mediated genomic instability. (**B**) The resolution of R-loops involves the recruitment of the RNA/DNA helicase, SETX, which is recruited to R-loops by SMN - defects in either cause motor neuron cell death.

In the past decade, it has become apparent that transcription is a primary source of DNA damage, and that RNA processing factors are important guardians of the genome (Dutertre *et al.*, 2016). RNA/DNA hybrids form during transcription when the nascent RNA transcript anneals to the template DNA strand, displacing the complementary DNA strand, and yielding a three-stranded nucleic structure known as an R-loop (Aguilera and Garcia-Muse, 2012). R-loop accumulation leads to genomic instability (Huertas and Aguilera, 2003; Li *et al.*, 2005; Tuduri *et al.*, 2009; Sollier *et al.*, 2014), and thereby couples RNA processing to the regulation of genomic stability. This was first observed when yeast mutant strains lacking members of the THO complex (mammalian equivalent of the transcription and export, TREX, complex) displayed increased numbers of R-loops and DNA breaks (Huertas and Aguilera, 2003). Similar results were observed after serine/arginine-rich splicing factor 1 (SRSF1) depletion in vertebrates (Li *et al.*, 2005), demonstrating that RNA processing factors are important regulators of R-loop homeostasis. Mechanistically, it is speculated that RNA processing factors prevent R-loop formation in a preventative fashion (Fig. 1A), by binding to the nascent RNA transcript and blocking R-loop formation (Chan *et al.*, 2014*b*). In follow-up studies, a separate class of transcription and export proteins, known as hnRNPs, were linked to the regulation of R-loops (Santos-Pereira *et al.*, 2013). HNRNP mutations cause ALS and FTD (Kim *et al.*, 2013), but whether these mutations induce R-loop-mediated genomic instability has not yet been reported.

A similar phenomenon has since been observed for the ALS-linked RNA processing factors, FUS and TDP-43 (Hill *et al.*, 2016). Depletion of either leads to an increase in DSBs, which are reduced by overexpression of the R-loop-specific nuclease, RNase H1. TDP-43 and FUS form damage-induced nuclear foci, which co-localize with RNA polymerase 2 and phosphorylated histone H2AX (γH2AX) (Hill *et al.*, 2016). These data suggest that TDP-43 and FUS function at the interface between transcription and DNA repair, likely by controlling R-loop levels. Based on this work, another interesting hypothesis is that TDP-43 proteinopathy may function similar to TDP-43 depletion, and induce R-loop-associated genomic instability. This would be of significance as 97% of ALS cases display TDP-43 proteinopathy (Neumann *et al.*, 2006; Ling *et al.*, 2013). Other ALS-mutant RNA processing factors exhibit similar cytoplasmic mislocalization (Kapeli *et al.*, 2017), suggesting that the nuclear-cytoplasmic shuttling of RNA binding proteins is an important disease mechanism. Indeed, defects in nucleocytoplasmic transport have emerged as an important pathological mechanism in C9orf72-ALS (Freibaum *et al.*, 2015; Jovicic *et al.*, 2015; Zhang *et al.*, 2015*a*). As well as the cytoplasmic accumulation of RNA processing proteins, the nuclear accumulation of mRNA has also been observed in C9orf72-ALS disease models (Rossi *et al.*, 2015), suggesting that improper RNA export is a consequence of dysfunctional RNA processing. An interesting avenue for future research would be to assess the association between nucleocytoplasmic transport defects, dysfunctional RNA processing, and the levels of R-loop-associated genomic instability.

In addition to R-loop protection via RNA processing factors, R-loops can be resolved by RNase H1, which degrades the RNA strand of RNA/DNA hybrids (Cerritelli and Crouch, 2009). A number of RNA/DNA helicases have also been implicated in the resolution of R-loops (Skourti-Stathaki *et al.*, 2011; Yuce and West, 2013; Hodroj *et al.*, 2017; Song *et al.*, 2017). For instance, the RNA/DNA helicase, SETX, has been linked to the resolution of R-loops (Skourti-Stathaki *et al.*, 2011; Yuce and West, 2013). Notably, autosomal dominant mutations in the *SETX* gene are known to cause a juvenile form of ALS, referred to as ALS4 (Chen *et al.*, 2004). In addition, autosomal recessive mutations in SETX can cause ataxia with oculomotor apraxia 2 (AOA2) (Moreira *et al.*, 2004). Disease mutations appear to occur within the helicase domain or within its N-terminal protein–protein interaction domain, suggesting either loss of helicase activity or loss of functional interactions is pathological.

Nonsense mutations in the *SMN1* gene lead to a juvenile form of motor neuron disease called spinal muscular atrophy (SMA) (Melki *et al.*, 1990). Recent evidence suggests that SMN is also important for the resolution of R-loops (Zhao *et al.*, 2016). Mechanistically, the tudor domain of SMN was shown to be a requisite for the recruitment of SETX to R-loops, and spinal muscular atrophy patient fibroblasts display increased levels of R-loops and DSBs (Zhao *et al.*, 2016). In addition, a mouse model of spinal muscular atrophy recapitulated these two hallmarks of genomic instability (Jangi *et al.*, 2017), suggesting that SMN is an important factor in the prevention of R-loop-driven genomic instability. These reports suggest that motor neuron cells may be particularly sensitive to perturbations in R-loop homeostasis, caused by defects in RNA binding proteins (Fig. 1B).

R-loops are enriched at GC-rich sites of the genome (Chan *et al.*, 2014*a*), which may occur because guanine-rich RNA: cytosine-rich DNA duplexes are thermodynamically more stable than the respective DNA: DNA duplex (Roy and Lieber, 2009). Susceptibility to R-loops is also increased by the presence of G-quadruplex structures (Duquette *et al.*, 2004; Aguilera and Garcia-Muse, 2012), which assemble in G-rich DNA sequences. The *C9orf72* repeat expansion has a high GC content and is known to form G-quadruplex structures (Haeusler *et al.*, 2014; Zhou *et al.*, 2015; Brcic and Plavec, 2017). Unsurprisingly, R-loops have been identified *in vitro* following the transcription of C9orf72 expansions (Haeusler *et al.*, 2014). *C9orf72* RNA also forms G-quadruplex structures, which sequester a number of RNA binding proteins (Cooper-Knock *et al.*, 2014). The sequestration of certain RNA processing factors may impede their function in R-loop prevention. In-line with this idea, cells expressing C9orf72 expansion constructs, as well as C9orf72-ALS post-mortem tissues, display increased R-loop levels (Walker *et al.*, 2017). The accumulation of R-loops in C9orf72 expansion expressing cells is a source of genomic instability, since overexpression of SETX reduces levels of DSBs. SETX overexpression also reduced cellular toxicity in C9orf72 expansion-expressing cells (Walker *et al.*, 2017), indicating that excessive R-loop accumulation is able to promote cell death in cellular models of ALS. Taken together, recent evidence suggests that RNA misprocessing, in the context of ALS, results in R-loop accumulation and the formation of toxic DSBs.

### Double-strand break repair signalling

Following DSB induction, the MRN (Mre11, Rad50, Nbs1) complex form foci at the break site (Williams *et al.*, 2007), which promotes ataxia-telangiectasia mutated (ATM) autophosphorylation at serine 1981 (Uziel *et al.*, 2003). ATM then sets into motion the DNA damage response: histone H2AX is phosphorylated on serine 139 by ATM, ATR and DNA-PK (Rogakou *et al.*, 1998; Blackford and Jackson, 2017). Phosphorylated H2AX recruits the mediator of DNA damage checkpoint 1 (MDC1) (Hartlerode and Scully, 2009), inducing the recruitment of the E3 ubiquitin ligase, RNF8 (Kolas *et al.*, 2007), which associates with phosphorylated TQ sites of MDC1 and attaches mono-K63-linked ubiquitin chains to H2A(X) (Huen *et al.*, 2007; Kolas *et al.*, 2007; Mailand *et al.*, 2007). RNF168 associates with these mono-ubiquitylated chains, and further catalyses the mono- and poly-ubiquitylation of H2A(X) (Stewart *et al.*, 2009). The ubiquitylation of H2A(X) by RNF168 is mandatory for the recruitment of 53BP1 and BRCA1 to sites of DSBs (Wang and Elledge, 2007), whose recruitment dictates DSB repair choice towards non-homologous end-joining or homologous recombination, respectively (Ciccia and Elledge, 2010). Interestingly, 53BP1 recruitment to sites of DSBs is also required for concentrating ATM into nuclear foci at DSBs (Noon *et al.*, 2010), suggesting a reciprocal relationship between ATM and 53BP1 in mediating DSB repair.

### Defective autophagy leads to defective DNA repair signalling

P62 plays a central role in selective autophagy and the UPS (Katsuragi *et al.*, 2015), which require its LC3-binding (LCB) and ubiquitin-associated (UBA) domains. While p62 resides predominantly in the cytoplasm, it is also found in the nucleus (Wang *et al.*, 2016). P62 binds to misfolded protein aggregates and targets them for degradation (Bjørkøy *et al.*, 2005). During p62-mediated autophagy, p62 is degraded along with its substrate, therefore autophagy self-regulates the cellular levels of p62 (Ichimura *et al.*, 2008). In turn, the accumulation of protein aggregates or defects in autophagy leads to p62 accumulation (Korolchuk *et al.*, 2009; Wang *et al.*, 2016). Recently, a seminal discovery demonstrated that excessive p62 acts as a negative regulator of DSB repair signalling (Wang *et al.*, 2016). Initially, it was observed that p62 depletion leads to increased levels of nuclear ubiquitylated protein foci, while p62 overexpression induced the opposing effect and similarly decreased levels of H2A ubiquitylation. Overexpression of p62 also prevented damage-induced 53BP1 foci formation, which could also be recapitulated by treating wild-type cells with the autophagy inhibitor, 3-methyladenine (3-MA). Importantly, 3-MA did not induce DSB repair defects in p62 depleted cells, demonstrating that autophagy inhibition leads to DSB repair impairment in a p62-dependant manner.

**Figure 2:**
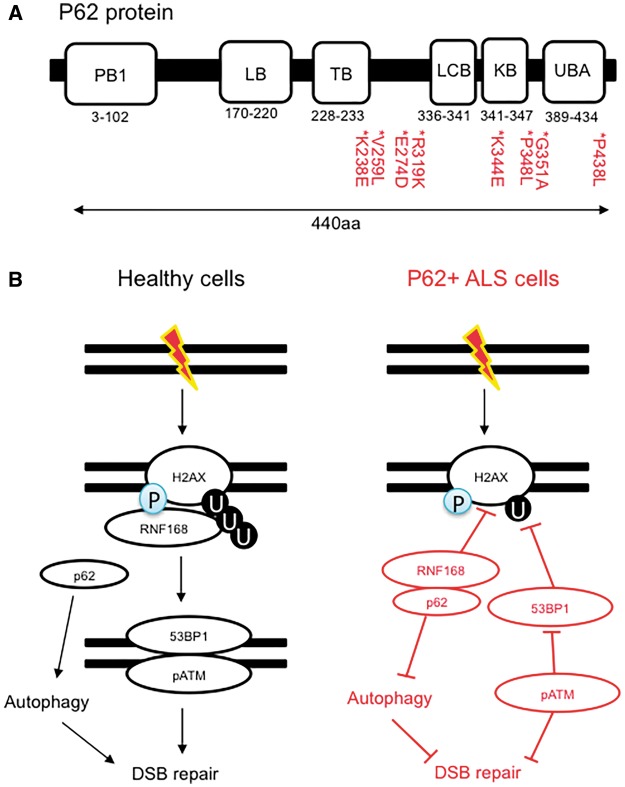
**Defective autophagy leads to defective DNA repair signalling.** (**A**) A schematic depicting the structure of p62 highlighting ALS, FTD, and IBMPFD missense mutations. (**B**) Autophagy maintains functional DNA repair by preventing p62 accumulation. In the context of ALS, where p62 accumulates, RNF168-mediated H2A ubiquitylation (U) is perturbed, leading to impaired DSB repair and genomic instability.

Biochemical assays demonstrated that p62-mediated DSB repair inhibition was a consequence of RNF168 sequestration, an E3 ubiquitin ligase that functions to recruit 53BP1 to DNA breaks (Stewart, 2009). P62 binds to the MIU (motif interacting with ubiquitin) domain of RNF168, which is important for its E3 ligase activity (Wang *et al.*, 2016). On the other hand, the LIM-binding (LB) domain of p62 (residues 170–220) mediates RNF168 sequestration and DSB repair inhibition. These data demonstrate that the accumulation of p62 impairs the DNA damage response. Interestingly, ALS-causing p62 mutations can occur in or around the LC3 domain of p62 (Fig. 2A), causing autophagy defects and the accumulation of mutant p62 (Rubino *et al.*, 2012; Goode *et al.*, 2016). An interesting future experiment would be to determine whether ALS-causing mutations in p62 cause DSB repair defects.

### C9orf72 repeat expansions lead to genomic instability

Excitingly to the field of ALS, p62 accumulation is a hallmark feature of patient CNS tissues (Blokhuis *et al.*, 2013), including C9orf72-ALS (Troakes *et al.*, 2012), suggesting that this hallmark pathology may be associated with defects in DNA repair. Recently, it was demonstrated that C9orf72 repeat expansions cause defective DNA repair, as indicated by decreased H2A ubiquitylation, impaired 53BP1 and phosphorylated ATM (pATM) repair foci, and increased levels of DSBs (Walker *et al.*, 2017). RNF168-mediated H2A ubiquitination is important for regulating 53BP1 and ATM mediated repair (Noon *et al.*, 2010). In turn, RNF168 overexpression or p62 depletion restored DSB repair and reduced levels of DSBs in C9orf72 expansion expressing cells (Fig. 2B). These data demonstrate that defective autophagy, indicated by p62 accumulation, is a driver of genome instability in C9orf72-ALS disease models (Walker *et al.*, 2017). It is likely that this phenomenon also occurs in non-C9orf72 ALS subtypes that exhibit p62 accumulation.

Increased DNA damage response activation in C9orf72-ALS patients has been corroborated by two separate reports (Farg *et al.*, 2017; Walker *et al.*, 2017), which demonstrated that motor neurons from C9orf72-ALS patients display increased levels of _ϒ_H2AX phosphorylation. It was demonstrated by immunohistochemistry that phosphorylated levels of ATM were increased in C9orf72-ALS motor neurons (Farg *et al.*, 2017), though the authors did not observe or quantify pATM repair foci. Importantly, defective ATM signalling in C9orf72-ALS was found to be the result of RNF168 dysfunction, a process that promotes the focal concentration of ATM but not its overall activation (Noon *et al.*, 2010). Using western blotting from unfractionated spinal cord homogenates, it was reported that C9orf72-ALS patient tissues have increased levels of 53BP1 protein (Farg *et al.*, 2017). Importantly, the extraction protocol used by the authors does not appear to extract the chromatin-bound fraction of tissue lysates, where 53BP1 protein engaged in DNA repair is enriched. Thus, neither pATM immunohistochemistry nor 53BP1 western blotting are indicative of DNA repair capacity. A crucial future experiment is to assess levels of 53BP1 repair foci in C9orf72-ALS tissues, particularly in those cells that are enriched with the hallmark accumulation of p62, and to repeat the immunoblotting experiments to specifically examine the levels of chromatin-bound 53BP1.

In other research, the expression of C9orf72-related poly-glycine-arginine dipeptide repeats (poly-GR DPRs) was shown to cause increased levels of genomic instability (Lopez-Gonzalez *et al.*, 2016). Unlike previous work, poly-GA DPRs did not induce γH2AX foci formation, though they were not quantified. A similar increase in DSBs was observed in C9orf72-ALS induced pluripotent stem cell-derived motor neurons, when compared to non-ALS controls (Lopez-Gonzalez *et al.*, 2016). Treatment with antioxidants partially decreased levels of nuclear DNA damage, suggesting that elevated levels of reactive oxygen species (or defective repair of reactive oxygen species-induced lesions) contributes to genome instability in C9orf72-ALS. In summary, there is now a wealth of evidence showing that C9orf72 expansions cause genomic instability. This is likely driven by multiple sources: (i) RNA processing dysfunction and R-loops accumulation; (ii) defects in autophagy and p62-mediated inhibition of DNA repair; and (iii) mitochondrial perturbations and the generation of reactive oxygen species.

### Familial ALS proteins are double-strand break repair players

**Figure 3:**
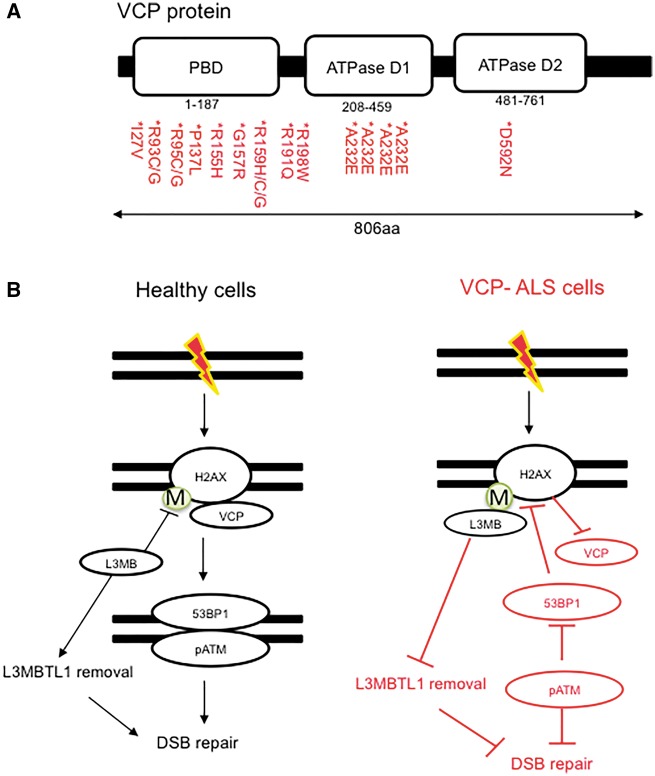
**The familial ALS protein VCP promotes DNA repair.** (**A**) A schematic depicting the structure of VCP protein highlighting ALS, FTD, and IBMPFD missense mutations. (**B**) VCP functions to catalyse the removal of ubiquitylated proteins from chromatin, including L3MBTL1. The removal of L3MBTL1 from methylated (M) histone H2A(X) permits 53BP1 recruitment to DSBs, enabling DNA repair by non-homologous end-joining. Loss of VCP leads to impaired removal of L3MBTL1, leading to impaired non-homologous end-joining and genomic instability.

ALS-linked genomic instability can therefore arise, indirectly, due to deficits in RNA processing and/or cellular clearance pathways. In addition to these mechanisms, proteins commonly mutated in ALS have been shown to play a more direct role in DSB repair. For example, VCP is a novel DSB repair player and its depletion leads to elevated levels of DSBs (Acs *et al.*, 2011; Meerang *et al.*, 2011). Mechanistically, VCP functions to catalyse the removal of lysine 48 ubiquitin conjugates at DNA damage sites, which is obligatory for the recruitment of DNA repair factors. As in C9orf72-ALS, VCP depletion impairs the recruitment of the DNA repair factor, 53BP1, to sites of DNA damage (Acs *et al.*, 2011; Meerang *et al.*, 2011), suggesting a common theme by which 53BP1-mediated DNA repair is defective in ALS (Fig. 3A). In the absence of VCP, L3MBTL1 is constitutively bound to H4K20me2, a histone residue that is recognized by the tudor domain of 53BP1, thereby blocking 53BP1 recruitment (Acs *et al.*, 2011). VCP is therefore required to remove L3MBTL1 and unmask H4K20me2 residues, permitting 53BP1 recruitment (Fig. 3B).

VCP has been further implicated in DSB repair, by removing lysine 48 conjugated Ku70/80 rings from DNA (van den Boom *et al.*, 2016). Failure to remove Ku70/Ku80 rings perturbs DSB repair, leading to genomic instability. How ALS causing mutations in VCP affect DSB repair, however, is not yet clear. Some ALS-causing VCP mutations affect the ATP binding domain of VCP (Fig. 3A) (Schuetz and Kay, 2016). Since the ATPase activity is required for its function in regulating DSB repair (Meerang *et al.*, 2011; van den Boom *et al.*, 2016), it is likely that these mutations result in an impairment of DNA repair signalling. Furthermore, due to its additional role in cellular degradation pathways, VCP mutations cause p62 accumulation in patient samples (Ayaki *et al.*, 2014), and may therefore cause additional defects in DNA repair through p62-dependant processes. Taken together, these data indicate that VCP has a direct role in repairing DNA breaks.

**Figure 4:**
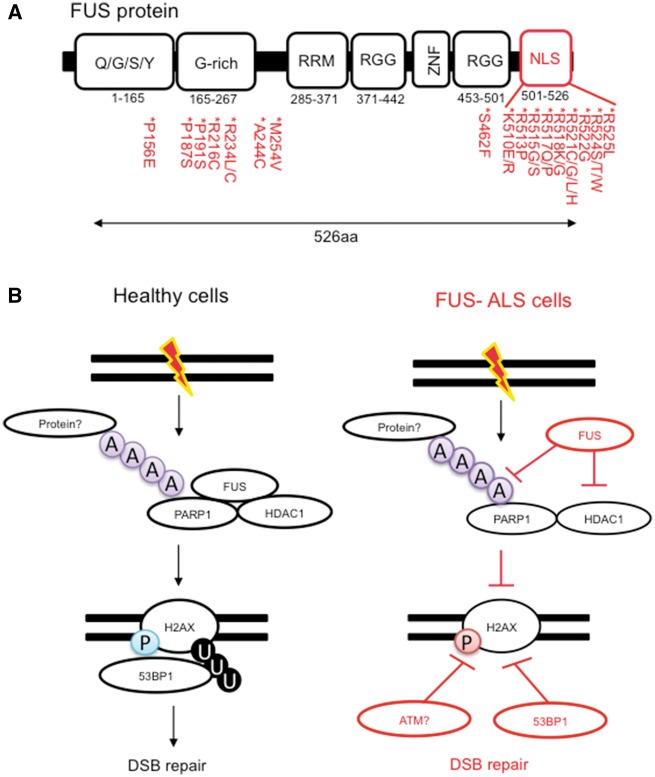
**The familial ALS protein FUS promotes DNA repair.** (**A**) A schematic depicting the structure of the FUS protein with ALS missense mutations highlighted. (**B**) FUS functions to regulate the DNA damage response through interactions with HDAC1 and PARP1. These interactions are important for H2AX phosphorylation (P) and 53BP1-mediated DSB repair. Loss of FUS or ALS-linked mutations leads to defective DSB repair and genomic instability.

In addition to VCP, the ALS-linked protein FUS has also been implicated in repairing DNA breaks (Fig. 4A) (Wang *et al.*, 2013; Rulten *et al.*, 2014). FUS depletion leads to impaired 53BP1 foci and increased DNA damage (Wang *et al.*, 2013), bearing striking similarities to what has been observed following the expression of C9orf72 expansions and the depletion of VCP (Acs *et al.*, 2011; Meerang *et al.*, 2011; Walker *et al.*, 2017). FUS recruitment to DSBs is facilitated by interactions with HDAC1, and ALS-linked FUS mutations impede this interaction and perturb DSB repair (Fig. 4A and B) (Wang *et al.*, 2013). Finally, FUS-ALS patient tissues display increased DNA damage, as measured by ϒH2AX levels in neuronal cells. In a separate study, FUS recruitment to DNA damage was dependent on PARP-1 catalytic activity (Rulten *et al.*, 2014). It was also shown that FUS binds directly to PAR chains and that ALS-causing mutations decrease this interaction (Rulten *et al.*, 2014). Taken together, these data indicate that FUS is a member of the DNA damage response, and indicate that ALS-associated defects in FUS lead to defects in DNA repair processes.

More recently, it was shown that mutation variants of the NIMA-related kinase 1 (*NEK1*) gene confer susceptibility to ALS (Kenna *et al.*, 2016). NEK1 has been implicated in the repair of DSBs by homologous recombination (Spies *et al.*, 2016), functioning to phosphorylate Rad54 and ensuring the adequate removal of chromatin-associated Rad51 filaments, which is required for homologous recombination completion. Mutations in C21orf2*,* an interactor of NEK1, are also associated with ALS (van Rheenen *et al.*, 2016). Moreover, its depletion similarly leads to defects in homologous recombination (Fang *et al.*, 2015). Many canonical homologous recombination factors, such as BRCA1 and BRCA2, have been linked to the processing of R-loops (Bhatia *et al.*, 2014; Hatchi *et al.*, 2015; Tan *et al.*, 2017; Zhang *et al.*, 2017). We therefore would like to propose that NEK1 and C21orf2 play a role in controlling R-loop homeostasis, which may explain at least in part their association with ALS.

## Heterochromatin: protecting from R-loops but perturbing DNA repair

Eukaryotic DNA is highly organized, typically the cell houses active genes in open euchromatic DNA, while non-transcribed or repressed genetic material is housed in tightly bound heterochromatin. In addition to promoting genomic instability, R-loops have been shown to alter the chromatin landscape by increasing levels of heterochromatic DNA. Of note, R-loop accumulation increases the levels of H3S10P (Castellano-Pozo *et al.*, 2013), a heterochromatic histone mark usually associated with mitosis. More recently, the same group demonstrated that R-loop-driven H3S10P formation is a requisite for subsequent genomic instability (Garcia-Pichardo *et al.*, 2017). Whether levels of H3S10P are therefore perturbed in in ALS samples is yet to be tested.

It was also demonstrated that H3K9 methylation is enriched in response to R-loop accumulation (Castellano-Pozo *et al.*, 2013). Increased H3K9 methylation has been reported in C9orf72-ALS (Belzil *et al.*, 2013; Walker *et al.*, 2017), which might be driven by the aberrant accumulation of R-loops in ALS (Haeusler *et al.*, 2014; Walker *et al.*, 2017). Interestingly, this mark has also been associated with Friedreich’s ataxia and fragile X syndrome, whereby trinucleotide repeat expansions were shown to cause R-loop-dependent H3K9me2 deposition (Groh *et al.*, 2014). H3K9 methylation marks and R-loops are also enriched at GC-rich transcriptional pause sites (Skourti-Stathaki *et al.*, 2014). It is postulated that H3K9 methylation may play a protective role, since abolishment of all H3K9 methylation induces massive R-loop accumulation and genomic instability in *Caenorhabditis elegans* (Zeller *et al.*, 2016). These data couple R-loop accumulation to the presence of heterochromatic DNA. An attractive model for this association is that the formation of repressive heterochromatin serves to reduce transcriptional activity and protect from dangerous R-loop-mediated instability.

While heterochromatin may protect the cell from the toxic effects of R-loops, the accumulation of heterochromatin represents an obstacle for DNA repair. Work in the Jeggo laboratory demonstrated that heterochromatic DSBs display slower repair kinetics than euchromatic breaks (Goodarzi *et al.*, 2008; Noon *et al.*, 2010). Interestingly, ATM and 53BP1 are particularly required for the repair of heterochromatic DSBs (Dimitrova *et al.*, 2008) and the inhibition of either impairs the resolution of heterochromatic DNA breaks (Goodarzi *et al.*, 2008; Noon *et al.*, 2010). Mechanistically, ATM-mediated phosphorylation of KRAB-associated protein 1 (KAP1), a *bona fide* heterochromatic protein, was shown to release KAP1 from heterochromatin and enable DSB repair (Goodarzi *et al.*, 2008). 53BP1 was subsequently shown to be upstream of ATM-mediated phosphorylation of KAP1 (Noon *et al.*, 2010), acting to concentrate active ATM at sites of DSBs. It is conceptualized that the phosphorylation and mobility of KAP1 promotes local chromatin decondensation that enables DNA repair factor access to the break site. In line with this, the genetic inhibition of KAP1 improves heterochromatic DSB repair kinetics in ATM inhibited cells (Goodarzi *et al.*, 2008). As such, heterochromatic DSBs are particularly challenging to cells because they require additional chromatin remodelling prior to repair.

Paradoxically, recent evidence suggests that the presence of local heterochromatic DNA is also a requirement for DSB repair signalling (Sun *et al.*, 2009; Kaidi and Jackson, 2013; Burgess *et al.*, 2014). The heterochromatic histone mark, H3K9me3, is required for the damage-dependent activation of the acetyltransferase KAT5 (Sun *et al.*, 2009). KAT5 activation, in turn, is required for the activation of ATM (Kaidi and Jackson, 2013). More recently, it was shown that chromatin compaction activates the DNA damage response, specifically proteins that occur upstream in DSB repair signalling, such as γH2AX (Burgess *et al.*, 2014). Importantly, excessive chromatin compaction impaired the completion of DNA repair and caused reduced cellular viability (Burgess *et al.*, 2014). These data suggest that, while heterochromatin is an important aspect of initial DSB repair signalling, it is ultimately a barrier to the proper completion of DNA repair.

As such, the careful organization of DNA into euchromatin and heterochromatin is critical to cellular health. Evidently, perturbations in these processes have been linked to human disease, such as premature ageing (Zhang *et al.*, 2015*b*) and ataxia telangiectasia (Li *et al.*, 2012, 2013). Chromatin relaxation, via HDAC inhibition or enhancer of zest homologue 2 (EZH2) depletion, was able to reduce the cellular and neurological phenotype of ataxia telangiectasia mouse models (Li *et al.*, 2012, 2013), suggesting a pathological role for excessive heterochromatin in the neurological aspect of the disease. Elevated levels of the heterochromatin have also been reported in C9orf72-ALS samples (Belzil *et al.*, 2013; Walker *et al.*, 2017). Interestingly, HDAC inhibition was able to reduce DSBs and cellular toxicity in models of C9orf72-ALS (Walker *et al.*, 2017). These data implicate excessive heterochromatin formation as a pathological feature of ataxia telangiectasia and ALS, highlighting the potential of HDAC inhibitors as a means to prevent neuronal cell death. Whether excessive heterochromatin is the cause or consequence of defective ATM, a feature in both disorders, is yet to be determined. Nevertheless, these data offer a plausible therapeutic approach and a new insight into the role of ATM in controlling chromatin compaction.

### Defective ATM signalling provides an unexpected link between amyotrophic lateral sclerosis and ataxia telangiectasia

Defective ATM signalling provides a molecular link between ALS and ataxia telangiectasia. Curiously, a recently characterized murine model of ataxia telangiectasia displays motor neuron cell loss (Quek *et al.*, 2017), indicating that motor neurons may be particularly sensitive to perturbations in ATM signalling. As well as its canonical role in DSB repair, ATM signalling is important for the repair of topoisomerase I (TOP1) cleavage complexes (Das *et al.*, 2009). In non-replicating cells, TOP1 cleavage complexes are primarily repaired by TDP1 (El-Khamisy *et al.*, 2005), and TDP1 mutations cause the neurodegenerative disease spinocerebellar ataxia with axonal neuropathy (SCAN-1) (Takashima *et al.*, 2002). ATM phosphorylates TDP1 (Das *et al.*, 2009) to enhance TOP1 cleavage complex resolution. In addition to TDP1 and ATM, a scaffold protein, XRCC1, further facilitates the repair of TOP1 cleavage complexes (Das *et al.*, 2009). Recently, *XRCC1* mutations were identified in a patient with cerebellar ataxia (Hoch *et al.*, 2017). As well as promoting neurodegeneration in man, *Atm, Tdp1* and *Xrcc1* deletion leads to the accumulation of TOP1 cleavage complexes in mice (Katyal *et al.*, 2007, 2014; Alagoz *et al.*, 2013). Exposure to camptothecin, which specifically induces TOP1 cleavage complexes, also leads to neurodegeneration in mice (Katyal *et al.*, 2014), further highlighting the pathogenic consequences of TOP1 cleavage complex in neurons.

Interestingly, TOP1 cleavage complexes are also increased in cells expressing C9orf72 repeat expansions (Walker *et al.*, 2017). This is in-line with the observation that C9orf72 expansions impair ATM signalling. Since defective ATM signalling was a consequence of p62 accumulation, p62 accumulation in other ALS subtypes likely drives the formation of TOP1ccs. Recently, it was shown that ATM is activated by R-loops, independently from the formation of DSBs (Tresini *et al.*, 2015). ATM activation by R-loops enhances the mobility of the spliceosome, ultimately leading to mis-splicing and the retention of introns within mRNA transcripts (Tresini *et al.*, 2015). The function of ATM kinase activity in mediating the cellular response to R-loops is yet to be elucidated, though it is tempting to speculate that these data represent yet another ATM-mediated repair pathway. In line with this idea, restoring ATM signalling in cells expressing C9orf72 expansion expressing cells was able to reduce R-loop levels (Walker *et al.*, 2017). The function of ATM signalling in the regulation of R-loop homeostasis, in both health and disease, represents a fruitful avenue for future research.

### Why does defective ATM signalling caused by C9orf72 expansions not predispose to cancer or immunodeficiency?

One of the primary features of ataxia telangiectasia is cancer predisposition, namely lymphomas (Taylor *et al.* 2004). The ATM deficiency in C9orf72-ALS patients, however, does not appear to predispose to cancer. While ataxia telangiectasia and C9orf72-ALS share defective ATM signalling, the nature and origin of this defect are fundamentally different. The ATM defect in C9orf72-ALS originates from the impaired ability to degrade misfolded protein aggregates, which leads to p62 accumulation and RNF168 inhibition (Walker *et al.*, 2017). RNF168 deficiency impairs 53BP1-mediated sustained signalling of ATM, a non-canonical pathway complementing the canonical ATM activation cascade initiated by the MRN complex (Noon *et al.*, 2010). Notably, RNF168 dysfunction causes another human disease typified by radiosensitivity, immunodeficiency and learning difficulties called RIDDLE syndrome, which similarly does not appear to be linked to cancer predisposition (Stewart *et al.*, 2007). In ataxia telangiectasia, oncogenesis is thought to arise as a consequence of defective DSB repair by homologous recombination and defective cell-cycle checkpoint activation (Cremona and Behrens, 2014). RIDDLE cells, however, display normal levels of homologous recombination and functional ATM-mediated cell-cycle checkpoint, potentially protecting patients from cancer (Stewart *et al.*, 2007). Thus, one possible explanation for the absence of cancer in C9orf72-ALS is the retention of functional homologous recombination and ATM-mediated cell cycle checkpoint, akin to RIDDLE. While this provides a plausible explanation for the absence of cancer in C9orf72-ALS, it does not explain the apparent absence of immunodeficiency, a hallmark of defective non-homologous end-joining in RIDDLE cells. In dividing cells, misfolded protein aggregates are asymmetrically distributed into daughter cells, leaving one of the daughter cells aggregate-free (Rujano *et al.*, 2006). Therefore, even if p62 accumulation occurs in ALS lymphocytes, non-homologous end-joining dysfunction would only persist for a single cell cycle and thus the toxic effect of protein aggregation is inherently ‘diluted’ in cycling cells. Indeed, protein aggregates typically impact non-dividing neuronal cells (Ross and Poirier, 2004; Taylor *et al.*, 2004; Bjørkøy *et al.*, 2005; Rujano *et al.*, 2006; Stewart *et al.*, 2007; Cremona and Behrens, 2014). We favour the latter explanation in which the magnitude of toxic effects caused by protein aggregation is higher in non-cycling compared to cycling cells, providing an explanation for the apparent absence of immunodeficiency in C9orf72-ALS. The extent of protein aggregation and the preset threshold at which non-cycling cells can tolerate may also explain the selective vulnerability of specific neuronal populations to the toxic effects of protein aggregations.

### The vulnerability of neurons to oxidative DNA damage

Despite representing only 2% of total body mass, one-fifth of all oxygen is consumed by the brain (Nicholls and Budd, 2000). Because they metabolize a high proportion of inhaled oxygen, neurons are likely to be highly susceptible to damage from reactive oxygen species. The primary free radical that is formed during cellular respiration is O_2_^•−^ (superoxide) (Murphy, 2009), which is dismutated into hydrogen peroxide (H_2_O_2_) by superoxide dismutase 1 (SOD1). SOD1 mutations cause ALS (Rosen *et al.*, 1993) and result in high levels of oxidative stress (Tsang *et al.*, 2014). Reactive oxygen species generated by mutant SOD1 expression induce breaks in the mitochondria and nucleus of human cells (Tsang *et al.*, 2014; Chiang *et al.*, 2017). These breaks also require processing by TDP1 (Ben Hassine and Arcangioli, 2009; Chiang *et al.*, 2017), and mutant SOD1 expression in a TDP1 knock-out background was toxic (Chiang *et al.*, 2017). Thus, ALS-causing SOD1 mutations lead to the over-accumulation of reactive oxygen species, causing oxidative DNA damage requiring TDP1-mediated repair. Elevated levels of reactive oxygen species have also been reported in other ALS subtypes, including C9orf72-, FUS-, TDP-43- and sporadic-ALS disease models (Smith *et al.*, 2017).

### Therapeutic potential of PARP inhibition and NAD(+) replacement strategies

Several lines of independent evidence have linked ALS to genomic instability. Perhaps the most important question, though, is how can we utilize this knowledge to modulate the disease? Cerebellar ataxias are commonly caused by mutations in DNA repair proteins (Madabhushi *et al.*, 2014). It has become apparent that cells defective in DNA repair processes typically display PARP1 hyperactivation (Lee *et al.*, 2009; Krenzlin *et al.*, 2012; Fang *et al.*, 2014; Scheibye-Knudsen *et al.*, 2014; Hoch *et al.*, 2017), as greater demand becomes bestowed upon PARP1-mediated repair pathways. In-line with this idea, *XRCC1* knock-out mouse models display PARP1 hyperactivation (Lee *et al.*, 2009; Hoch *et al.*, 2017). Deletion of the *Parp1* gene in *Xrcc1* knock-out mice is able to prevent neurodegeneration, indicating that the overactivation of PARP1 is a neurotoxic consequence of defective DNA repair (Hoch *et al.*, 2017).

PARP enzymes catalyse the addition of poly-ADP-ribose (PAR) chains onto glutamate or serine residues of target proteins (Bai, 2015). Cellular pools of NAD+ are the donor molecule, which PARP enzymes catalyse during this process. It is postulated that PARP1 hyperactivation leads to the overconsumption of NAD+, underpinning the neurodegeneration observed in XRCC1-null mice (Hoch *et al.*, 2017). Indeed, several neurological diseases that are characterized by defective DNA repair, including ataxia telangiectasia, are associated with PARP1 hyperactivation and low levels of cellular NAD+ (Krenzlin *et al.*, 2012; Fang *et al.*, 2014, 2016; Scheibye-Knudsen *et al.*, 2014). These data suggest that pharmacological inhibition of PARP1 may be protective. However, current PARP1 inhibitors have been shown to induce the trapping of PARP1 onto chromatin (Pommier *et al.*, 2016). PARP trapping would likely cause neurotoxicity by inducing DNA lesions and blocking transcriptional elongation, making current PARP inhibitors unsuitable as disease-modifying agents for treating neurodegeneration. Next generation PARP1 inhibitors, which block the enzymatic action of PARP1 while preventing its trapping onto chromatin, may offer a promising therapeutic potential for ALS and related diseases.

As well as PARPs, cellular NAD+ is further consumed by another family of enzymes called sirtuins (SIRTs) (Vassilopoulos *et al.*, 2011). SIRTs function as NAD+ dependent lysine deacetylases, which regulate a number of biological processes, including metabolism and DNA repair (Michan and Sinclair, 2007). SIRT1, for instance, promotes DSB repair by deacetylating and in turn activating HDAC1 (Dobbin *et al.*, 2013). SIRT1 activity appears to be impeded by PARP1 hyperactivation (Cantó *et al.*, 2015). In addition, NAD+ replenishment has been shown to reactivate SIRT1 and prevent neurodegeneration (Scheibye-Knudsen *et al.*, 2014), thereby compensating for PARP1 hyperactivation. An attractive model for these data is that compromised DNA repair leads to PARP1 hyperactivation, resulting in the depletion of NAD+ levels, limiting NAD+ dependent SIRTs. The repletion of NAD+ therefore restores SIRT1 function, reduces genomic instability, and ultimately prevents neurodegeneration. As such, NAD+ replenishment strategies represent a promising new strategy for neurological diseases characterized by DNA repair dysfunction. Indeed, the repletion of NAD+ has been shown to positively modulate the disease course in models of ataxia telangiectasia, Cockayne syndrome and xeroderma pigmentosium (Krenzlin *et al.*, 2012; Fang *et al.*, 2014, 2016; Scheibye-Knudsen *et al.*, 2014).

Given the emerging role of dysfunctional DNA repair, particularly defective ATM-mediated repair, in ALS it may be possible that similar strategies would confer therapeutic benefits. Indeed, motor neurons are particularly sensitive to perturbations in cellular NAD+ levels. Deletion of intracellular nicotinamide phosphoribosyltransferase (iNAMPT), the rate-limiting enzyme involved in NAD+ biosynthesis, leads to a motor neuron disease phenotype in mice (Wang *et al.*, 2017). It is striking that the specific targeting of NAD+ levels causes a motor neuron disease phenotype. Moreover, iNAMPT protein levels were significantly reduced in ALS patient spinal cord tissues (Wang *et al.*, 2017), reinforcing the notion that NAD+ replenishment could offer new therapeutic benefit in ALS.

### The selective sensitivity of motor neurons to genomic instability

Motor neurons, like Purkinje cells that degenerate in ataxia telangiectasia and related disorders, are among the largest cells of the human body. Their large size is probably linked to high levels of cellular respiration, elevated levels of transcription and in turn, a high dependency upon functional cellular clearance pathways to maintain cellular homeostasias. Unsurprisingly, defective RNA processing, impaired cellular clearance pathways, and dysfunctional mitochondrial respiration are linked to the pathogenesis of ALS—all of which drive genomic instability. Defective RNA processing leads to genomic instability due to the formation of R-loops, while dysfunctional mitochondrial respiration leads to oxidative damage. Autophagy defects lead to genomic instability due to the over accumulation of p62 and the suppression of DSB repair signalling. The high metabolic, transcriptional, and autophagic activity of motor neurons may render these cells particularly vulnerable to perturbations in these, intrinsically linked, cellular processes.

## Abbreviations

ALS: amyotrophic lateral sclerosis
DSB: double-stranded break
FTD: frontotemporal dementia
